# Automating Glycan Assembly in Solution

**DOI:** 10.1021/acscentsci.2c01043

**Published:** 2022-09-30

**Authors:** Yong Wu, Yunyan Qiu, Yuanning Feng, J. Fraser Stoddart

**Affiliations:** † Department of Chemistry, Northwestern University, 2145 Sheridan Road, Evanston, Illinois 60208, United States; ‡ School of Chemistry, University of New South Wales, Sydney, New South Wales 2052, Australia; § Stoddart Institute of Molecular Science, Department of Chemistry, Zhejiang University, Hangzhou 310027, China; ∥ ZJU-Hangzhou Global Scientific and Technological Innovation Center, Hangzhou 311215, China

Scientists’ understanding of structure–function relationships^1,2^ involving carbohydrates—one of the essential biomacromolecules in living systems—is not so detailed as those relating to DNA/RNA and proteins. Consequently, carbohydrates are far from widely used in the development of new therapeutics and diagnostics or in materials science. The bottlenecks hampering the growth of the field of glycoscience lie, not only in the lack of robust sequencing methods, but also in the difficulties associated with obtaining pure and well-defined glycans in adequate quantities from natural sources on account of their heterogenicity. While the chemical synthesis of glycans provides a promising solution to this conundrum, it represents a long-standing challenge for synthetic chemists, especially for those without a specialized knowledge of carbohydrate chemistry. Glycans can be linear or branched, and each glycosidic linkage is associated with a stereogenic center that requires perfect regio- and diastereo-control during every coupling step in a synthesis. As a result, glycan synthesis^3^ entails the skillful deployment of a bewildering array of protecting groups, extensive optimization of coupling conditions, and tedious separation of intermediates: all are time-consuming and labor-intensive.

The emergence of machines, such as steam engines, that are capable of performing work, has released human beings from the ordeal of repeated labor and has shaped the face of modern society. Similarly, the invention of automated synthesizers—employing both chemical and enzymatic reactions—for the construction of DNA/RNA and peptides/proteins in an efficient and rapid manner, has revolutionized modern science during the past few decades. These automated chemical syntheses are usually based on solid-support chemistry in which a growing oligonucleotide or peptide is anchored covalently to an insoluble resin, allowing their facile purification by filtration and washing, in addition to the release of sequence-defined oligomers after linker cleavage. Applying analogous approaches to oligosaccharide assembly, however, has proved to be more challenging on account of the relatively low reactivity associated with glycosidic bond formation on conventional solid supports compared with the use of fast amidite-phosphoric diester formation for oligonucleotide synthesis and amide formation for peptide synthesis. Since the development of the first solid-phase oligosaccharide synthesizer by Seeberger et al.^4^ in 2001, several automated platforms—based on chemical and enzymatic glycosylations^5−7^—for glycan assembly have been introduced. Although these automated platforms have increased the efficiency of glycan assembly to a significant extent, challenges remain. They include, but are not limited to, (1) the use of a large excess of expensive glycosyl donors, (2) relatively limited substrate scope, (3) the difficulties in direct monitoring of the progress of reactions, and (4) the challenges associated with the scale-up of reactions.

**Figure 1:**
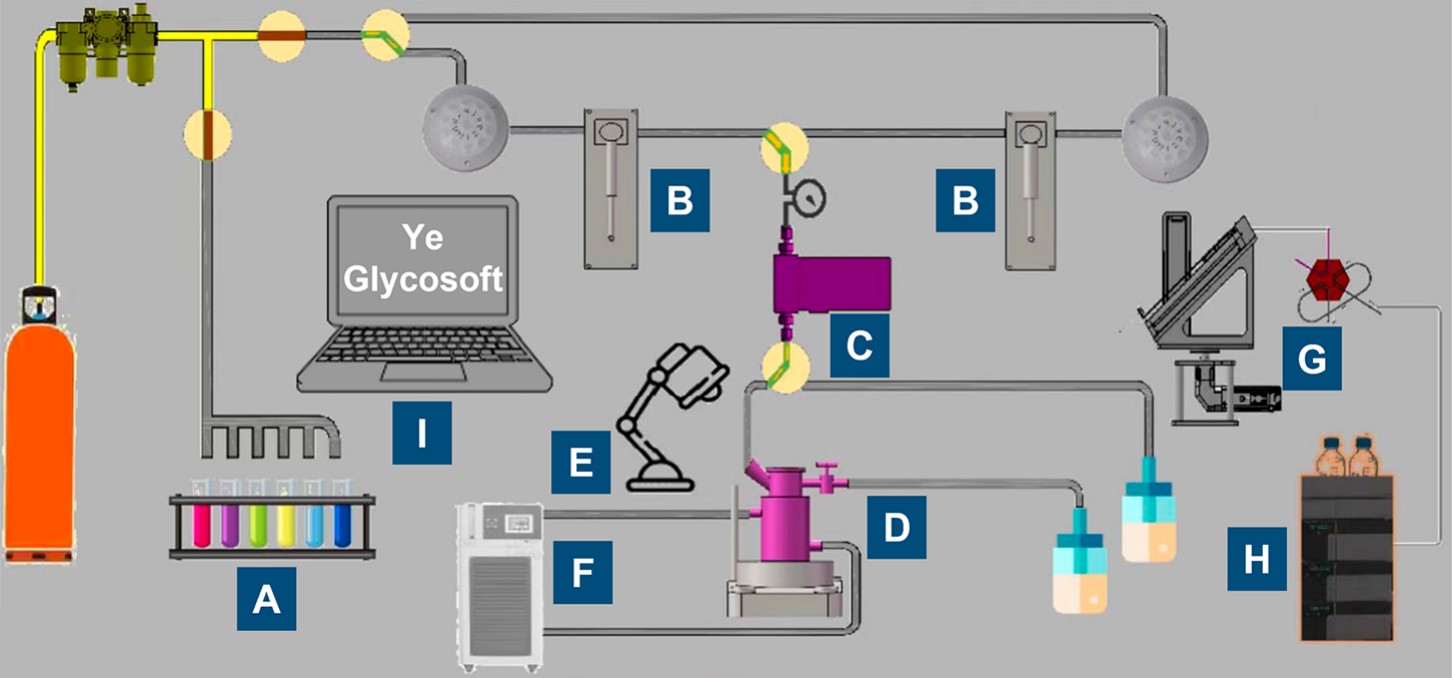
Schematic illustration of the automated glycan synthesizer. The synthesizer consists of four parts: (1) An automatic injection system, including **A**. Storage racks for glycosyl building blocks, reagents, and solvents. **B**. Syringe pumps, and **C**. A mass flow controller (MFC) for sample withdrawal and injection. (2) An automated synthetic system, including **D**. A three-jacketed reactor for chemical glycosylations. **E**. A mercury lamp for light-promoted activation of glycosyl donors, and **F**. A cryostat for temperature control. (3) An online monitoring system, including **G**. An automatic sampling module, and **H**. An analytical HPLC. (4) **I**. A programmable logic controller (PLC), which is controlled by a computer software called “Ye Glycosoft”.

**Figure 2:**
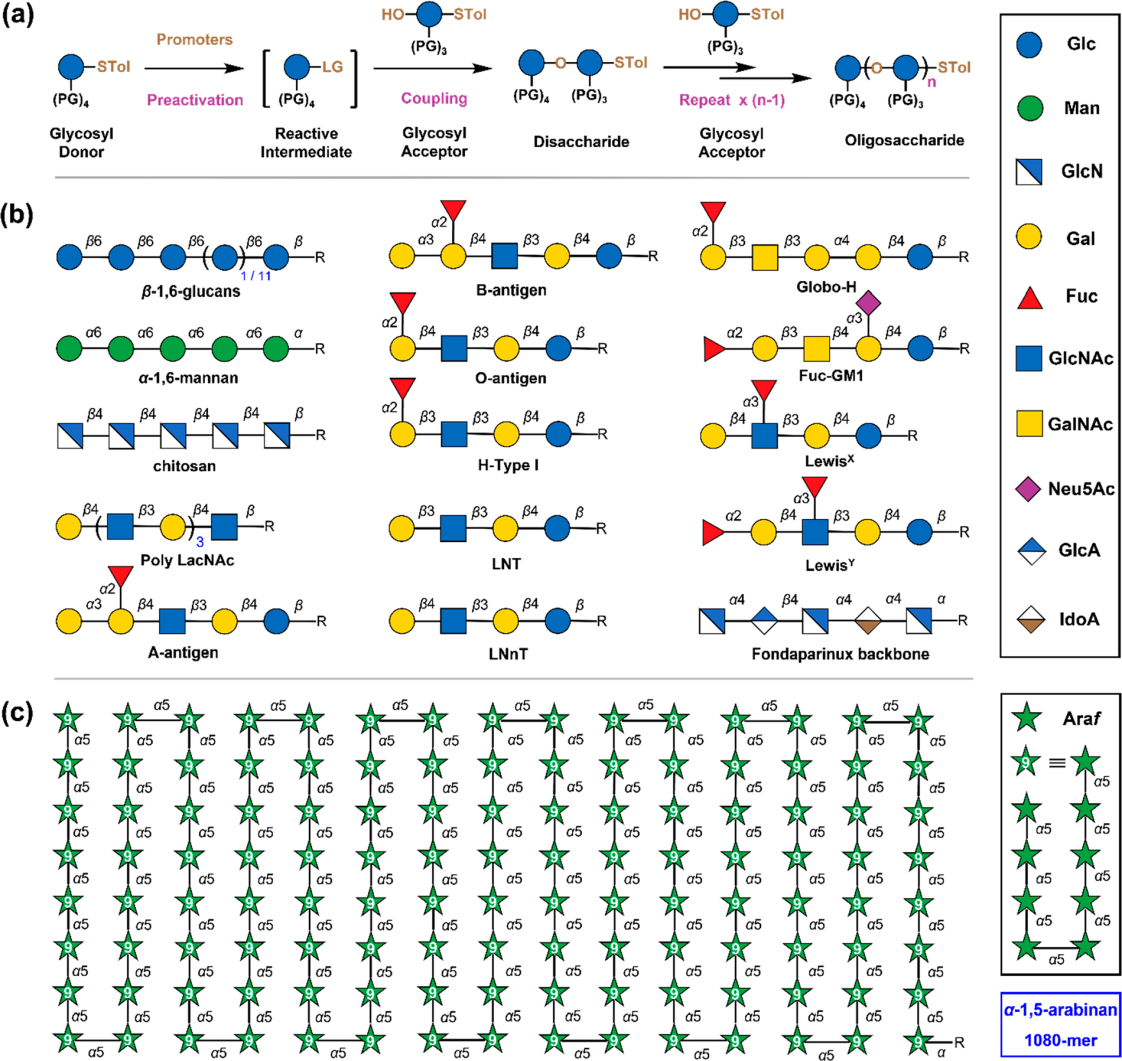
Automated glycan assembly in the solution phase. (a) The mechanism of the multicomponent, one-pot chemical glycosylations. A glycosyl donor is preactivated by promoters in the absence of a glycosyl acceptor to generate a reactive intermediate (preactivation), which can react with a glycosyl acceptor to produce a disaccharide (coupling). By repeating these preactivations and couplings, a sequence-defined oligosaccharide can be obtained. (b) Symbol nomenclature for glycans (SNFG) representation of oligosaccharides that have been assembled by the automated synthesizer. The protecting groups are omitted for the sake of clarity. (c) The SNFG representation of a 1080-mer α-1,5-arabinan produced by the synthesizer. Abbreviations: PG, protecting group; STol, *p*-methylphenyl sulfenyl; LG, leaving group; Glc, glucose; Man, mannose; GlcN, glucosamine; Gal, galactose; Fuc, fucose; GlcNAc, *N*-acetylglucosamine; GalNAc, *N*-acetylgalactosamine; Neu5Ac, *N*-acetylneuraminic acid; GlcA, glucuronic acid; IdoA, iduronic acid, Ara*f*, arabinofuranose.

Recently, Ye and co-workers^1^ have reported a dual-mode glycan synthesizer (Figure 1) based on the automation of solution-phase-based, multicomponent, one-pot chemical glycosylations^8^ (Figure 2a) in which several glycosyl building blocks are preactivated, using either chemical- or light-promoted protocols before reacting sequentially to produce sequence-defined oligosaccharides as the main products. This automated synthesizer, which (1) uses stoichiometric amounts of glycosyl building blocks and (2) monitors the progress of reactions directly employing online HPLC, can be carried out on a gram scale, thus overcoming some of the drawbacks of previously reported automated synthesizers. The broad scope of this new machine has been vindicated by the streamlined assembly of a wide variety of highly complex and structurally different oligosaccharides (Figure 2b), including protected β-1,6-glucans, an α-1,6-mannan, chitosan, poly-*N*-acetyllactosamine (poly-LacNAc), histo-blood group antigens including the A, B, O, and H-type I antigens, core structures of human milk oligosaccharides (HMOs) and glycosphingolipids (GSLs) including lacto-*N*-tetraose (LNT) and lacto-*N*-neotetraose (LNnT), tumor-associated carbohydrate antigens (TACAs) including Globo-H, Fuc-GM1, Lewis^X^ and Lewis^Y^, and the protected Fondaparinux (an anticoagulant).

In addition to the successful assembly of a library of linear and branched oligosaccharides, this automated synthesizer can execute the production of polysaccharides consisting of up to 1080 monosaccharide residues by employing a multiplicative synthetic strategy (Figure 2c). Although suffering from low reactivities and steric hindrance during large-fragment couplings, as well as difficulties in the global deprotection of 2161 protecting groups and purification of the final product, Ye and co-workers^1^ managed to accomplish the synthesis after several rounds of optimization of reaction and separation conditions. The fully deprotected 1080-mer α-1,5-arabinan (142.8 kDa) with 4320 stereogenic centers not only marks the longest and largest ever-made homogeneous polysaccharide in the history of carbohydrate chemistry—a bold step in comparison with the previous syntheses^9−11^ of large glycans using either manual or automated protocols—but also raises the chemical syntheses of biomacromolecules to a completely new technical level that reaches far beyond the records chalked up already in the production of polynucleotides^12^ (up to 200-mer) and polypeptides^13^ (up to 472-mer).

The present work, which constitutes the first example of synthesizing a homogeneous biomacromolecule with a four-digit monomer count, represents a milestone in state-of-the-art synthetic chemistry. Turning the clock back over half a century, one of us (J.F.S.) was involved in the analysis^14^ of naturally occurring polysaccharides (gum arabic) during his Ph.D. studies at Edinburgh in the 1960s at which time making disaccharides was a suitable research project for a Ph.D. candidate to undertake. It is more than surprising and encouraging for us to find out that Ye and co-workers^1^ are now able to demonstrate how far carbohydrate chemists can push the envelope in glycan synthesis, enabled by the ingenious design and invention of an automated solution-phase synthesizer. Besides the superiority of the protocol, another merit of the current automated platform is the potential to integrate other strategies^15−18^ employing one-pot transformations of carbohydrates in the most commonly used solution phase.

Looking to the future, with such enabling techniques in the toolbox for the efficient construction of oligo- and polysaccharides, one might ask: what can be achieved next? There are countless research opportunities to pursue in order to answer many fundamental questions in glycoscience. These questions include, but are not limited to, the following: (1) Can we make highly branched polysaccharides with a range of monosaccharide residues (neutral and charged) having different (α and β) anomeric configurations? (2) Can polysaccharides form complex tertiary and quaternary structures as proteins do? (3) Can polysaccharides execute more biological functions other than those recognized currently? (4) Can we manipulate the function of polysaccharides by tailor-made synthesis? (5) Can polysaccharides find practical applications in the development of new functional materials? The present research, together with the imagination and enthusiasm of scientists of different persuasions, will undoubtedly help answer these questions and add yet another dimension to our knowledge about the structure–function relationships of carbohydrates, which will, in turn, advance applications in carbohydrate-related nanotechnology, not to mention the biomedical and materials sciences.
